# The Cancer Paste

**Published:** 1913-03

**Authors:** 


### ABSTRACTS.

---

The Cancer Paste has recently been revived and has been given a respectable status. A. Zeller, *Münch. Med. Woch.*, Aug. 20 and 27, 1912, reports 57 authentic cases, 44 cured, 3 dead, 10 still under treatment. After cleansing the surface with benzine, a paste containing arsenic and cinnabar is thickly applied, covered with collodion or a regular aseptic dressing and renewed every week or two. One-half gram each of sodium and potassium silicate, are administered t. i. d. and are continued for a year after apparent cure. (Note—As local treatment of cancer of this fashion is limited to external growths, easily diagnosed promptly, it seems best to adhere to extirpation except in neglected, inoperable cases. At the same time, the results of chemic and radiant treatment are suggestive for the future. The administration of soluble glasses in so large dose and for so long a time, leads to speculation as to the possible results of combination with lime.)

